# Mānuka Oil—A Review of Antimicrobial and Other Medicinal Properties

**DOI:** 10.3390/ph13110343

**Published:** 2020-10-26

**Authors:** Cynthia Mathew, Wubshet Tesfaye, Phil Rasmussen, Gregory M Peterson, Andrew Bartholomaeus, Manab Sharma, Jackson Thomas

**Affiliations:** 1 Faculty of Health, University of Canberra, Canberra 2617, Australia; Cynthia.Mathew@canberra.edu.au (C.M.); Wubshet.Tesfaye@canberra.edu.au (W.T.); g.peterson@utas.edu.au (G.M.P.); a.bartholomaeus@uq.edu.au (A.B.); Manab.Sharma@canberra.edu.au (M.S.); 2 Phytomed Medicinal Herbs Ltd., Auckland 1026, New Zealand; hil@phytomed.co.nz; 3 Tasmanian School of Pharmacy, University of Tasmania, Hobart 7001, Australia; 4 Faculty of Medicine, University of Queensland, Brisbane 4072, Australia

**Keywords:** mānuka oil, *Leptospermum scoparium*, antimicrobial, antibacterial, antiparasitic, medicinal, therapeutic properties

## Abstract

Mānuka oil is an essential oil derived from *Leptospermum scoparium*, a plant that has been used by the indigenous populations of New Zealand and Australia for centuries. Both the extracted oil and its individual components have been associated with various medicinal properties. Given the rise in resistance to conventional antibiotics, natural products have been targeted for the development of antimicrobials with novel mechanism of action. This review aimed to collate available evidence on the antimicrobial, anti-parasitic and anti-inflammatory activities of mānuka oil and its components. A comprehensive literature search of was conducted using PubMed and Embase (via Scopus) targeting articles from database inception until June 2020. Chemical structures and IUPAC names were sourced from PubChem. Unpublished information from grey literature databases, Google search, targeted websites and Google Patents were also included. The present review found extensive in vitro data supporting the antimicrobial effects of mānuka oil warrants further clinical studies to establish its therapeutic potential. Clinical evidence on its efficacy, safety and dosing guidelines are necessary for its implementation for medical purposes. Further work on regulation, standardization and characterization of the medicinal properties of mānuka oil is required for establishing consistent efficacy of the product.

## 1. Introduction

Mānuka (*Leptospermum scoparium*), also known as kahikatoa, red mānuka and tea tree, belongs to the *Myrtaceae* plant family and is found throughout New Zealand and Australia [1]. Commonly grouped under ‘tea trees,’ which also includes *Camellia sinensis, Kunzea ericoides*, *Leptospermum petersonii* and *Melaleuca alternifolia*, these species have been used by the Māori, the Aboriginals and early European settlers as topical preparations for wounds, cuts, sores and skin diseases and as inhalations for colds and fevers. Interest in the medicinal properties of mānuka, particularly mānuka honey, has grown over the last 30 years. There is also a growing interest in the volatile oil due to its potential application as a medicinal agent or cosmetic ingredient [2,3]. Currently, there has not been a critical review on the efficacy and potential applications of mānuka oil while there have been extensive reviews on Mānuka honey and its medical applications. We aim to bridge this gap through this review, providing an up-to-date summary of recent developments in our understanding of the medicinal activities of mānuka oil as well as its clinical efficacy—and toxicity profile.

## 2. Search Strategy

Literature search was conducted using PubMed and Embase (via Scopus) targeting articles from database inception until June 2020. Key terms like ‘*Leptospermum scoparium*’ and ‘antimicrobial’ or ‘antibacterial’ or ‘anti-inflammatory’ or ‘antiviral’ were tailored for individual databases to perform the search (a full search strategy is summarized in Appendix A). The retrieved articles were filtered manually to exclude duplicates and then screened using titles, abstracts and full-text articles based on our objectives. Also, bibliographies of included studies were reviewed to check for articles missed during the main search. Chemical structures and IUPAC names were obtained from PubChem. Information was also gathered from grey literature databases, Google search, targeted websites and Google Patents.

### 2.1. Chemical Composition

Mānuka oil (CAS 219828- 87-2) is a volatile essential oil derived from the foliage, bark and seeds of *Leptospermum scoparium* plants of the *Myrtaceae* family of trees and shrubs [4]. A clear liquid with an aromatic odor is produced by a steam distillation process from plants harvested mostly in the autumn, summer and spring, with a yield ranging from 0.2–1%, depending on seasonal and geographical factors [5,6] (also commercial data held by Phytomed Medicinal Herbs Ltd., Auckland, New Zealand). The oil is generally distilled using leaves and young stems, although oil has also been produced from the seeds [5,7]. The use of biowaste fertilizer has recently been reported to enhance oil production and content [8].

A large number of studies have examined the constituents of mānuka oil, which vary depending on the source of the oil [7,9,10] as well as the plant chemotype and season of collection [11]. Overall, 100 components were identified from 16 commercial samples of mānuka oil, of which 51 components made up 95% of the content [7]. The major components of commercially available mānuka oils are reported to be leptospermone (0.8–19.4%), calamenene (2.5–18.5%), δ-cadinene (0.9–6.9%), cadina-1,4-diene (0.1–5.9%), flavesone (0.7–5.8%), cadina-3,5-diene (3.0–10.0%), α-copaene (4.3–6.5%) and α-selinene (1.3–5.0%) (Table 1) [7,9,11,12,13,14]. Chemotypes found in the East Coast of the North Island of New Zealand tend to contain higher levels of β-triketones than oil sourced from other regions [11]. The compartmentalization of β-triketones and grandiflorone within oil glands inside mānuka leaves may defend the plants against mānuka’s herbicidal activity [15]. There are also some types of *L. scoparium* that are high in α-pinene (21.5%) [11], though the majority of plants possess lower levels of this monoterpene (0.6–11%) [7,9,10,11].

Variation of the components in mānuka oil was found both between and within natural populations of *L. scoparium* [7,11,16]. Higher levels of triketones (known to have strong antibacterial properties) were predominant in samples from the East Cape population. Higher levels of the monoterpenes, α- and β-pinene, were present in the Northland populations [16]. Samples from *L. scoparium* grown in Australia had higher monoterpene levels and almost no triketones compared to those from New Zealand [16]. Mānuka oil from New Zealand was shown to have low concentrations of monoterpenes in comparison to kanuka oil (75% α-pinene) and negligible amounts of terpinen-4-ol and 1,8-cineole predominant in Australian tea tree oil [7]. In general, mānuka oils from different geographic locations around New Zealand can be simplified into 3 basic chemo-type groups based on the ratio of the monoterpenes:sesquiterpenes:ß-triketones [29]. These include triketone-rich in the East Cape (marketed as Manex™), monoterpene-, linalool- and eudesmol-rch in Nelson (Kaiteriteri™) and monoterpene- and pinene rich in Canterbury [5,9,16,30]. A varied chemotype in mānuka grown in South Africa in comparison to New Zealand-grown was shown by van Vurren [31]. The age of the plant also plays a role in the nature of the essential oil, with higher sesquiterpene content in young plants and a mixed amount of monoterpenes and sesquiterpenes in mature ones [9]. 

The lack of consistency in constituents is a common problem with natural extracts and a source of inconsistency in medicinal properties [32]. Though mānuka oil is listed under the New Zealand Inventory of chemicals (CAS 219828- 87-2), ECHA (CAS 223749-44-8) and EINECS (425-630-7), further research into regulation, standardization and characterization of the medicinal properties from varying origins is required.

### 2.2. Medicinal Properties of Mānuka Oil

Mānuka grows abundantly throughout New Zealand and has been a part of traditional Maori medicine for a variety of applications. The bark of the tree has been used for the treatment of skin diseases, as a sedative and as a mouthwash. The leaves are boiled in water for treating colds or crushed and applied to ease itching and scabs, while the leaves have been used as tea, as a febrifuge and for pain relief. The seeds were used for treating dysentery and diarrhea [33,34]. Contemporary data, prominently from in vitro studies, clearly demonstrates a broad spectrum of antibacterial, antifungal, anti-parasitic/insecticidal, anti-inflammatory, antiviral and spasmolytic activity.

### 2.3. Antibacterial Activity

The antibacterial activity of mānuka oil is the most characterized, being effective against both Gram-positive and Gram-negative bacteria (Table 2). These effects are variable depending on the type and source of the oil, with fractions containing triketone constituents seeming particularly active [10].

### 2.4. Mechanism of Action

The antibacterial activity of mānuka oil is relatively more pronounced against Gram positive bacteria than Gram negative bacteria (Table 2). The exact mechanism of the antibacterial effects in Gram positive bacteria are unknown but cell lysis of Gram-positive cells suggests disruption of the bacterial cell membrane is an important component of the mechanism. Treatment with 1.5% (*v/v*) of mānuka oil for 4 h induced morphological changes and cell lysis in methicillin-resistant *Staphylococcus aureus* (MRSA), while treatment with a high dose (3% *v/v*) completely disrupted the cells [35]. The β-triketone content are suggested to be responsible for this activity [30]. In contrast treatment with mānuka oil against Gram negative bacteria, such as *E. coli*, caused mild alterations in morphology at low doses and higher concentrations (6% *v/v*) were required for antibacterial effects [12,35]. The basis of the difference is unknown but this may be due to limited diffusion across the lipopolysaccharide-based capsule covering the outer membrane of Gram negative bacteria [35]. 

### 2.5. Gram Positive Bacteria

The efficacy of mānuka oil is both dose- and time-dependent. Treatment with a 10% solution of mānuka oil in DMSO was equally effective as the same concentration of kanuka oil against *Staphylococcus aureus, Staphylococcus sobrinus and Staphylococcus mutans* (Minimum Inhibitory Concentration (MIC) = 0.048% (480 µg/mL); the highest dilution at which no growth was observed) [36] (Table 2). A time to kill assay against the same strains showed 100% inhibition on treatment with either 10% mānuka oil or kanuka oil required as low as 5 s and as high as 900 s [37]. Another study determined the MIC value ranging between 0.13–0.25% against *S. sobrinus* strains and 0.25% against *S. mutans* [38]. Higher concentrations of essential oils are reported in the case of other antibacterial medicinal products against the same oral pathogens—tea tree (1%), eucalyptus (1%), lavender (>1%) and rosemary (>1%) [38] (Table 2). 

Mānuka oil exhibits strong bacteriostatic effects against different strains of *Staphylococcus*, including antibiotic-resistant strains (Table 2). Treatment with 2% (*v/v*) mānuka oil, kanuka oil or triketones in Tween 80 had limited bactericidal effects at 240 min post-treatment (using death a kinetics assay) [30]. Mānuka oil has also exhibited strong activity against different strains of *Staphylococcus pseudintermedius,* often contributory to skin and ear infections in dogs [39]. These included methicillin-resistant strains, with MICs being around 2% (*v/v*) for both resistant- and antibiotic-sensitive isolates [39]. Inhibition of *S. pseudintermedius* biofilm formation was also reported in the same study [39]. Exposure of various strains of *S. aureus* to subinhibitory concentrations of mānuka oil has also been found to significantly inhibit their ability to produce enterotoxins, an effect not observed after treatment with oregano and marjoram essential oils [36,40]. 

Activity against *Staphylococcus epidermidis* and *Propionibacterium acnes*, pathogenic bacteria responsible for acne in humans, has also been reported [41,42]. Interestingly, this study also found mānuka oil to show the highest likelihood amongst a range of different essential oil combinations, of being involved in synergist interactions against *S. epidermidis* [41]. Kim et al. and Wu (2011) have shown reduction in acne and bactericidal effects (MIC = 0.211% *w/v* or 2.11 mg/mL; MBC = 0.25% *w/v* or 2.5 mg/mL) of mānuka oil against *P. acnes* respectively [42,43].

Impressive effects of mānuka oil have been found against pathogenic bacteria associated with biosolid soil contamination, with significant growth inhibition of *C. perfringens* and *L. monocytogenes* [44]. The EC_50_ = 0.07% and 23.3% (environmental effect concentration is the dose required to reduce pathogen growth by 50%) for *C. perfringens* and *L. monocytogenes,* respectively, on treatment with concentrated mānuka leaf extract for 24 h [44].

Specific components or fractions of mānuka oil, such as leptospermone (and its derivatives, grandiflorone and myrigalone A), have also been identified to possess antibacterial activity [19,45]. Leptospermone had the strongest inhibitory effect against foodborne Gram positive bacteria, such as *Listeria monocytogenes, S. aureus and S. intermedius* (Table 2), using both dilution assay and agar diffusion assay (≥30 mm inhibition zone on treatment with 1 and 2 mg/disc) [19]. Leptospermone and its derivative 1,2,3-cyclohexanetrione-1,3-dioxime had strong inhibitory effects against intestinal bacteria, *Clostridium difficle* and *C. perfringens* (inhibitory zone ranged between 20 and >30 mm on treatment with 2 or 5 mg/disc) [19]. The derivative 1,2,3-cyclohexanetrione-1,3-dioxime had a strong inhibitory effect against *Bifidobacterium breve* and *B. longum* (≥30 mm inhibition zone on treatment with 5 mg/disc) but leptospermum had no effect on these strains. Neither fractions were effective against *Lactobacillus casei*, a non-pathogenic probiotic [19]. 

Two other components of typical mānuka oil, grandiflorone (MIC = up to 0.0032% or 32 μg/mL) and myrigalone A (MIC = 0.0064% or 64 μg/mL), have also been identified as active against vancomycin-resistant *Enterococcus faecalis* (VRE) and methicillin-resistant *S. aureus* (MRSA) and may therefore provide potential treatments for infection with these bacterial species [45]. 

### 2.6. Gram Negative Bacteria

A comprehensive study of the effects of 60 different essential oils on the growth of *Helicobacter pylori* (*H. pylori*) found mānuka oil to be the seventh most effective antibacterial when diluted in propylene glycol (500 µg/mL after 1 h and 40 µg/mL after 24 h) [46]. Mānuka oil derived from the North Island of New Zealand displayed significant antibacterial activity against 20/20 *Listeria monocytogenes* strains, whereas more so than that of mānuka oil from the South Island was effective against (0/20 *Listeria monocytogenes* strains) [28]. Higher levels of β-triketones found in chemotypes growing in the North Island of New Zealand, are likely to be contributory to these differences [11]. 

Investigation of the growth inhibitory effect of an oil from mānuka seeds against *Escherichia coli* showed it to be ineffective according to Jeong et al. (2009) while it was effective for Prosser et al. (2014). Based on calorimetric growth experiments using *E. coli* K12 C600, Jeong et al. (2009) showed that treatment with different doses of mānuka oil dissolved in Tween 80 (up to 4% *v/v*) had a dose-dependent effect in reducing bacterial growth but was not as effective as treatment with *Melaleuca alternifolia* oil [47]. On the other hand, Prosser et al. (2014) showed treatment with a very high dose of concentrated mānuka oil inhibited the growth of *E. coli* 0157 (EC_50_ = 27.8%). Similar doses were required to inhibit the growth of *Salmonella typhimurium*. In comparison, treatment with 0.597% of concentrated mānuka oil was sufficient to retard the growth of *C. jejuni* [44]. This study proposed the potential applications to improve polluted soils and adjacent waterways, through planting mānuka or including mānuka oil when biosolids are applied to the soil to help prevent bacterial contamination [44,48].

Mānuka-derived leptospermone has strong antibacterial activity against Gram negative foodborne bacterial pathogens, *Salmonella typhimurium*, *Shigella flexneri* and *Shigella sonnei*, with MICs ranging from 23.6 to 69.7 μg/mL [49]. These data suggest that leptospermone may be useful clinically or as a natural food preservative, as it is able to inhibit the growth of harmful gut and foodborne bacteria while displaying no significant effect on beneficial bacterial species [49]. 

Activity against various Gram negative, antibiotic-resistant bacterial isolates from dogs with otitis externa, has also been reported recently for mānuka. These included both antibiotic-sensitive and multidrug-resistant clinical isolates of *Pseudomonas aeruginosa, Escherichia coli, Klebsiella pneumoniae spp. pneumoniae* and *Proteus mirabilis* [50]. MIC and MBC values (minimum bactericidal concentrations) of mānuka oil alone were ≥1% *v/v* and ≥2%, respectively [50].

Mānuka oil was the most effective against periodontopathic bacteria in comparison to tea tree oil, eucalyptus oil, lavandula oil and romarinus oil. Mānuka oil was effective at very low concentration (MIC = 0.03%) against the oral pathogens *A. actinomycetemcomitans* (MBC = 0.13%)*, P. gingivalis* (MBC = 0.06; 0.03 for ATCC 53977) and *F. nucleatum* (MBC = 0.03%). By comparison, higher MIC values very observed for other antibacterial essential oils such as tea tree (0.06–0.5%), eucalyptus (0.13–0.5%), lavender (0.25–1.0%) and rosemary (0.5–1.0%) against the same oral pathogens [38]. 

### 2.7. Synergistic Effects

Combining mānuka oil with Tris-EDTA enhanced the antibacterial effects against drug-resistant isolates from dogs with otitis externa, with MIC value ranging from 0.06% to 0.5% and MBC value ranging from 0.06 to 1% [50]. A placebo-controlled study of bacterial pyoderma in dogs examined the effects of combining systemic antibiotics with a topical spray composed of essential oils, including mānuka oil [51]. The results showed a significant improvement in skin healing with the combination of oral antibiotics and essential oil spray, compared to oral antibiotics alone [51]. 

Similar results highlighting the synergistic effects of mānuka oil were shown by Filoche (2005). The combined effect of chlorhexidine digluconate and a series of essential oils, including mānuka oil, found that mānuka oil inhibited the growth of two cariogenic bacteria, *S. mutans* and *Lactobacillus plantarum,* either as liquid cultures or as biofilms [52]. When mānuka oil and chlorhexidine were combined, the authors found an eight-fold reduction in the concentration of chlorhexidine required to achieve the same level of growth inhibition, suggesting the potential for novel anti-caries treatments [52]. 

Combined treatment with *Cananga odorata* and mānuka oil had MIC = 0.094% (0.94 mg/mL) and 0.060% (0.60 mg/mL) against acne-causing *Propionibacterium acnes* (ATCC 11827) and *Staphylococcus epidermidis* (ATCC 2223), respectively. In comparison, treatment with each oil individually required higher doses (Table 2) (Orchard, 2018). Combined treatment with mānuka oil and *Achillea millefolium* or *Citrus aurantium* var. *amara* flower (neroli) had MIC = 1.0 mg/mL and 2.0 mg/mL against *P. acnes* and *S. epidermidis,* respectively, in the same study [41].

### 2.8. Antifungal Activity

A summary of the antifungal effects of mānuka oil is given in Table 3. Although the exact mechanism of action is unknown, the essential oil from Melaleuca alternifolia (tea tree) has been shown to cause damage to the fungal cell wall and cytoplasmic membrane thereby altering the membranes properties and disrupting their functions. Melaleuca oil also causes thinning and distortion of the hyphal wall, causing cell wall disruption and formation of empty hyphal tips that create bud-like structures [54].

Moderate rate of inhibition of phytopathogenic fungi Phytophthora cactorum (28 × 103 mg/mL air), Cryphonectria parasitica (14 × 103 mg/mL air) and Fusarium circinatum (7 × 103 mg/mL air) was revealed fumigant assay [55]. When the fungi were exposed to mānuka oil as a fumigant, the inhibition rate was 50% against *P. cactorum* and 62% against F. circinatum; however, no inhibition was observed against C. parasitica [55]. 

The effect of mānuka oil on Aspergillus niger, A. ochraceous and F. culmorum was assessed in another study, which found wide variation in the antifungal activity depending on the source of mānuka oil; the mānuka oil sample from the South Island of New Zealand had greater antifungal activity against A. ochraceus and F. culmorum than mānuka oil from the North Island [28]. The fungicidal activity of mānuka oil was assessed for a series of human fungal species, Malassezia furfur, Trichosporon mucoides, Candida albicans and C. tropicalis, with a MIC of 1.56% for M. furfur and T. mucoides and 3.13% for both Candida species [37]. Good activity against Candida species was also reported in a subsequent study [56].

### 2.9. Antiparasitic/Insecticidal Activity

Several studies have examined the antiparasitic activity of mānuka oil, for parasites with significant impact on plants, humans and animals (Table 4). The primary animal parasites under investigation have been poultry red mites (*Dermanyssus gallinae*), while the primary human parasites investigated have been scabies (*Sarcoptes scabei*), house dust mites (*Dermatophagoides farina* and *D. pteronyssinus*), stored product mites (*Tyrophagus putrescentiae*) and mosquitos (*Aedes aegypti*). The adsorption of the active ingredients in the oil are suggested to cause fumigant and/or contact toxicity in the parasites. Contact and fumigation assays are generally used to determine the antiparasitic efficacy of essential oils. The compound efficacy would therefore depend on their lipophilic nature, viscosity and vapor pressure. In contact bioassays, lipophilic and viscous compounds are commonly more effective as they penetrate the cuticle layer of the arthropod. In fumigation bioassays, the toxic compounds are inhaled via the respiratory system and are dependent on the vapor pressure exerted by the compound [57].

A study of the effect of mānuka oil on different life stages of *D. gallinae* suggests it is effective against both adult and juvenile mites but that it is not ovicidal for this parasite [58]. A study of the effect of mānuka oil on *D. gallinae* found the lethal concentration required to kill 50% of the parasites (LC_50_) during a 24-h period in a contact bioassay was ~0.05 mg/cm^3^ and 0.03 mg/cm^3^ [58]. Mānuka oil displayed a repelling effect on >75% of *D. gallinae* mites for up to 4 days after treatment of the mites contained in the Y-tube of an olfactometer and exposed to either fresh air in one arm of the Y-tube or air containing the volatile components of each essential oil in the other arm [59,62]. 

The effect of mānuka oil on a poultry beetle (*Tenebrio molitor),* a beneficial insect in a poultry system, was assessed along with a non-target organism. In this case, the mānuka oil had no significant effect on beetle mortality compared to the control (~15% vs. ~5%, respectively) [62]. Similar results for *T. molitor* exposure to mānuka oil were observed in another study [59]. In contrast, exposure of brine shrimp (*Artemia salina*, an organism commonly used in toxicity testing) to mānuka oil revealed a 90% mortality rate after exposure to ~0.05 mg/cm^3^, the same concentration as the LC_50_ for *D. gallinae* [59], indicating potential use of mānuka oil as a general acaricide may need careful consideration regarding the effect on non-target organisms. 

Mānuka seed oil appears to be effective against house dust (*D. farina* and *D. pteronyssinus*) and stored product mites (*Tyrophagus putrescentiae*), with a LD_50_ (dose required to kill half the members of a tested population after a specified test duration) values of 0.54, 0.67 and 1.21 µg/cm^2^, respectively, against the stated parasites; these values are 11.5–68.7 times more effective than DEET (*N,N*-diethyl-3-methylbenzamide), a common chemical treatment to control these mites [20]. Further analysis of the major components of mānuka seed oil indicated the main triketone, leptospermone, to be the most active component of mānuka oil in this study, with LD_50_ values of 0.07–0.15 ug/cm^2^ against *D. farina, D. pteronyssinus* and *T. putrescentiae*; these values represent 92.6–530.3 times the toxicity of DEET against the same organisms [20]. 

A study of the effects of essential oils on human scabies mites (*Sarcoptes scabei*) found mānuka oil to be moderately effective against the mites, with median lethal times of 30 min (± 7.5 min) after direct contact with a 10% manuka oil solution in paraffin oil. Vapor phase toxicity of mānuka oil was determined via fumigation assay, where mites were exposed to a filter paper treated with mānuka oil and mortality was checked every 5 min. Median lethal time for undiluted mānuka oil was 23 min (± 8.7 min) [14]. 

Insecticidal activity for mānuka oil has also been reported against *Drosophila suzukii*, a fruit fly pest which is a serious economic threat to soft summer fruit. Mānuka oil’s LD_50_ for contact toxicity was 0.60 μg/mL for males and 1.10 for females. Triketone components were shown to be contributory to these insecticidal effects [60]. 

Mānuka oil has also been used as a lure to attract *Xyleborus glabratus* (Redbay ambrosia beetle), an exotic wood-borer that transmits the fungal agent (*Raffaelea lauricola*) responsible for laurel wilt, which has had a severe impact on forest ecosystems in South-East United States [63,64].

Screening of essential oils for their toxicity against *Aedes aegypti* (L.) larvae found that mānuka oil containing calamenene and leptospermone as dominant constituents, exhibited strong larvicidal effects. This suggests potential applications for mānuka oil in mosquito vector control. These effects were enhanced when mānuka oil was combined with carvacrol or oregano oil [61] and with an emulsion made using amylose-N-1-hexadecylammonium chloride [21]. 

### 2.10. Anti-Inflammatory Effects

Experiments to assess the anti-inflammatory potential of mānuka oil found that THP-1 cells stimulated with lipopolysaccharide (LPS) and co-treated with 0.1–10% mānuka oil had significantly reduced release of TNF-α but there was no significant effect on the release of IL-4 [37]. This study found no cellular toxicity at an oil concentration of 10%, which contrasts with the results discussed in the toxicity section earlier. The diluent used in the study by Chen et al. was not specified and this discrepancy could be a result of diluting the lipophilic mānuka oil in aqueous cell culture media. Lis-Balchin et al. (2000) also showed the antioxidant effects of mānuka oil from the North- and South-islands of New Zealand were more consistent in comparison to kanuka oil [28]. 

### 2.11. Photo-Protective Effects

Solar ultraviolet (UV) radiation is the primary environmental factor causing skin damage and consequently premature aging. Kwon et al. (2013) evaluated mānuka oil (from Coast Biologicals Ltd., Auckland, New Zealand) for its effects against photoaging in UV-B-irradiated hairless mice. After 8 weeks of exposure to UVB radiation, mice that were treated topically with 10% mānuka oil experienced a reduction in typical UVB-related skin changes, such as skin thickening, appearance of wrinkles and loss of skin collagen [65]. These effects were associated with inhibition of loss of collagen fibers, reduction of epidermal hyperplasia, suppressed production of proinflammatory cytokines (IL-1β and TNF-α) and reduced macrophage infiltration, suggesting mānuka oil can inhibit UVB-associated inflammation in skin [65]. 

A nanoemulsion (particle size of 11.93 µm) containing mānuka oil (10% by weight) as the main component along with a Vitamin C derivative (ascorbyl tetraisopalmitate; 2% to 10% by weight) has been used in cosmetic formulations, including skin creams, lotions, essences and cosmetic powders [66]. The versatile formulation is effective as a whitening, anti-inflammatory agent and for wrinkle improvement [66]. Treatment for 9 weeks with the mānuka oil-Vitamin C derivative nanoemulsion in SKH-1 Hairless Mice exposed to artificial photoaging using UV-B irradiation showed increased thickness of the skin within 6 to 8 weeks post-treatment while the control showed no change.

### 2.12. Antiviral Activity

Herpes simplex viruses can cause cold sores (usually Herpes simplex virus type 1, HSV-1) or genital herpes (usually Herpes simplex virus type 2, HSV-2) and both can become chronic and recurrent infections sometimes resistant to antiviral drugs [67]. Pre-treatment with a β-triketone-rich mānuka oil has been reported to exhibit inhibitory effects on HSV-1 and HSV-2 [23,68,69]. RC-37 cells (monkey kidney cells) or the viruses were pre-treated with mānuka oil for one hour followed by inoculation. Pre-treatment of the virus with the oil significantly inhibited plaque formation in comparison to pre-treatment of the cells alone [23]. The IC_50_ against HSV-1 was 0.96 µg/mL (0.0001% *v/v*) and that for HSV-2 was 0.587 μg/mL (0.00006% *v/v*) [23]. Pre-treatment of host cells before viral infection reduced replication of HSV-1 by 41%. Treatment with flavesone and leptospermone alone, two characteristic triketone constituents of mānuka oil, had similar effects [23]. Another study reported pre-treatment of acyclovir-resistant isolates of HSV-1 and HSV-2 with 0.001% mānuka oil reduced the infectivity of the viruses by >99% [69,70]. The absence of viral inhibition in pre-treated cells suggests that the oil is likely to exert a direct antiviral effect on HSV before or during adsorption onto the host cells [23,69,70].

### 2.13. Spasmolytic Activity

Smooth muscle spasmolytic activity has been reported for mānuka oil in experiments on guinea pig ileum showing a dose-dependent inhibitory effect of mānuka oil on smooth muscle contractions [71]. A subsequent study of the components of mānuka oil showed α-terpineol and terpinene-4-ol both produce strong spasmolytic activity in guinea pig ileum, while in contrast, α- and γ-terpinenes displayed spasmolytic activity after an initial spasmogenic action [28]. A post-synaptic mechanism affecting cAMP to alter potassium channels in the muscle was suggested by the authors as the possible mode of action [28]. This activity was absent in kanuka oil and neither mānuka or kanuka oil used cGMP nor behave like potassium channel openers (seen in *Melaleuca* (tea tree) oil). 

Mānuka oil and its components were also reported to increase muscle tone in skeletal muscle, evaluating its absorption in chick biventer muscle and rat phrenic nerve diaphragm preparations [28]. In contrast, both mānuka oil and *Melaleuca* oil decreased uterine muscle contractions. Application of either oils showed a decrease in tension (via inhibition of twitch response on stimulating the skeletal muscle nerve) and a weighty increase in resting tone (indicating contracture) when the muscle was stimulated either directly or via the phrenic nerve. the components α-terpineol, α-terpinene and terpinene-4-ol showing a similar, significant decrease in the force of contractions [28]. The study suggests the use of these oils for aromatherapy, where they would aid as relaxants for those suffering from stress and anxiety. While these studies involved relatively high doses of a limited number of mānuka oil samples, whose origins and chemotypes were not well characterized, to isolated tissues in vitro [28]. The researchers highlighted caution against the use of mānuka oil as a relaxant during childbirth may be detrimental to the birthing process and should therefore be avoided in this situation [28]. This suggestion was based on similar properties exhibited by tea tree oil and other essential oils [28,72].

### 2.14. Safety and Tolerance

Mānuka oil, like other essential oils, has been classified as safe and tolerable for human use (CAS (US and EU) 223749-44-8; CTFA monograph ID 10572 and EINECS 425-630-7). However, the lack of clinical trials means there is limited data to inform dosing practices and toxicology profiles. The in vitro toxicity of mānuka oil and its main constituent, leptospermone, tended to vary with cell lines, concentrations tested and method of analysis (Table 5). Higher cytotoxicity was observed on treatment with mānuka oil in comparison to leptospermone alone. This suggests additional components of mānuka oil that makes it more toxic to cell lines. A study of mānuka oil in human umbilical vein endothelial cells (HUVEC) found treatment with a concentration of 0.2% of mānuka oil reduced cell viability by ~30% [38]. Given mānuka oil is lipophilic and cannot be diluted in aqueous cell culture media [69], it is possible the actual concentration of mānuka oil in contact with the HUVEC cultures was significantly less than assumed.

No signs of toxicity were observed on treatment with 10% active aqueous or oily phase of the cosmetic formulation for Campo Mānuka Oil Extract in fibroblast cells. In vitro organogenesis assay (Living Dermal Matrix (LDM)), a toxicity assay that closely mimics the effect of a substance on human skin, consists of skin cells in a 3D-construct made of collagen. The LDM test proved Campo Mānuka oil to be non-irritant with 99.4% cell viability after treatment with an undiluted sample in comparison to 100% propylene glycol (73%; a non-irritant) and 100% morpholine (6%; a moderate irritant) [73]. 

Unpublished data showed gel formulations containing >10% mānuka oil did not induce acute skin sensitivity in mice (unpublished data Phytomed Medicinal Herbs Ltd.). Acute toxicity was not observed (LD_50_ = 4612 g/kg) in mice after single oral administration of varying doses (500 mg/kg to 5000 mg/kg) of a patented formulation containing a mix of *Leptospermum scoparium* and *Kunzea ericoides* essential oils. The same formulation did not induce erythema or edema 3 and 7 days after a skin irritation test in epilated rabbits treated with 0.5 g of the product. Absence of percutaneous irritation was also demonstrated after treatment with 0.1 mL of 10% emulsion of the combined oil product onto the eyes of rabbits treated up to 7 days [74].

Product safety report on MELORA™ Mānuka oil (<5%) according to EC Regulation 1223/2009 detailed high skin tolerance and good cosmetic acceptability of the product. The formulation has a relatively high content of β-triketones, such as flavesone, isoleptospermone, leptospermone and grandiflorone. Acute toxicities based on the routes of administration were LD_50_ = 1061 mg/kg body weight and LD_50_ > 2000 mg/kg body weight for oral and dermal administration, respectively. No irritation was noted on testing the formulation on rabbit eye mucous membranes. There was no skin sensitization or genetic toxicity after a micronucleus test in TK6 Human lymphoblastoid cells and bacterial reverse mutation test in *Salmonella typhimurium* and *E. coli*. It is intended for external use, could cause irritations in the eye on direct contact and a patch test is suggested before use [6]. 

The safety evaluations for Campo™ Mānuka Oil Extract (10% concentrate in water or ceramide) formulation according to EC regulations described that the oil was non-toxic for dermal use and was edible in small quantities (oral LD_50_ = >9000 mg/kg body weight) after testing in rats. The formulation was classified as a non-irritant based on tests in vivo and in healthy human subjects. Patch tests in 50 healthy human subjects at doses from 0.5% to 100% showed satisfactory tolerance with no significant irritation reactions [73]. The irritation potential of a 10% solution of mānuka oil was tested on the chorio-allantoic membrane of chicken egg, using the Eyetex assay and Skintex assay. All three tests deemed the formulation as non-irritant. The product did not have any comedogenic effect on the skin, indicating that the product does not clog pores and is well tolerated on the skin [73]. 

Manuka oil sample pre-registered by the ECHA (EC Number: 434-370-3) was found to have LD_50_: 1.061 mg/kg body weight (95% CI: 722–1.557) for oral administration and LD50 >2000 mg/kg body weight. Low scores for erythema and edema were determined for skin irritation and eye irritation tests in vivo. 

Mānuka oil is an active ingredient in the TGA-listed product Kiwiherb Herbal Throat Spray (ARTG ID 337576), which contains a mixture of *Echinacea purpurea* (6%), honey (7%), mānuka oil (1 µL/mL), *Macropiper excelsum* var. *excelsum* (7%), propolis tincture (0.09 µg/mL and *Thymus vulgaris* (5%). The formulation is accepted in Australia by TGA for oral administration to reduce or relieve cold, cough, dry throat, mild throat inflammation, itchy throat and pharyngitis [75]. 

## 3. Future Directions

Mānuka oil has been extensively used in traditional medicinal preparations and its individual fractions, particularly *β*-triketone constituents, exhibit many bioactive properties. Yet its application in clinical medicine remains under explored. The emergence of new strains of pathogens, including bacteria, viruses, parasites and fungi, coupled with the continued rise in antibiotic resistance, warrants further study of the potential antimicrobial activity of essential oils as therapeutic agents for their control and eradication. In contrast to tea tree oil, there is limited evidence on the efficacy and safety of mānuka oil in terms of geographic origin, parts of the plant used, dilutions and variation among formulations. The elucidated mechanisms of action exerted by topical application of mānuka oil are general to those of essential oils. Further reproducible studies looking into the mechanistic and lethal action of mānuka oil as well as its fractions are required to accurately compare efficacy between formulations and sources of the oil. Future studies on the synergistic efficacy of mānuka oil with synthetic drugs or other essential oils could improve its efficacy against pathogen, for instance Gram negative bacteria. In vivo studies on the medicinal properties of mānuka oil could inform therapeutic and interventional clinical trials. Additionally, there is a clear need for standardization of manuka oil (e.g., the International Organization for Standardization [ISO] and Association Française de Normalization [AFNOR] for quality specification of key bioactive terpene components) to meet the growing needs of therapeutic grade product.

## 4. Conclusions

With emerging resistance to drug-based antimicrobial and anti-parasitic treatments, as well as a growing interest in alternative therapies, the extensive in vitro data supporting the medicinal properties of mānuka oil warrant further clinical studies to characterize its true therapeutic benefits. Commercialization and therapeutic application of mānuka oil requires extensive regulation and standardization of the oil and clinical evidence for its medicinal properties. The review highlights an underutilized essential oil with potential medical applications similar to tea tree oil and mānuka honey. Further characterization of antimicrobial activity of manuka oil will clearly improve its potential to become a clinically useful product. The current in vitro data suggest that manuka oil products may have efficacy in the treatment of many cutaneous skin infections. These data also provide a sound basis from which to proceed to in vivo and clinical work—which is critical in translating early laboratory findings into clinically useful outcomes.

Since the mechanism of action has implications for the selectivity and safety of antimicrobial agents, this issue is becoming increasingly important. Despite growing interest in the oil for therapeutic purposes, the current data remains limited, highlighting the need for further research to understand how manuka oil works. There is enormous potential to expand the manuka oil industry if claims about its medicinal properties are to be verified. Scientific validation regarding the antimicrobial activity of manuka oil may assist in accessing of international markets and the registration of the oil with national regulatory bodies. 

## Figures and Tables

**Table 1:** Chemical composition of Mānuka oil and known properties of each component.

Component	Percentage in Commercial Compositions	IUPAC Name	Known Properties	Ref.
α-pinene	Up to 21.5%	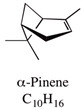	Reported to have antibiotic resistance modulation, anticoagulant, antitumor, antimicrobial, antimalarial, antioxidant, anti-inflammatory, anti-Leishmania, and analgesic effects in association with other essential oils.	[16,17]
Leptospermone	0.8–19.4%	6-isovaleryl-2,2,4,4-tetramethyl-1,3,5-cyclohexanetrione 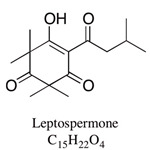	Herbicidal; antibacterial: treatment with 5–20 mg/disc of concentrate was effective against foodborne bacteria: *Listeria monocytogenes, Staphylococcus aureus and Staphylococcus intermedius* and three Gram-negative bacteria: *Salmonella typhimurium, Shigella flexneri and Shigella sonnei*	[18,19,20]
Calamenene	2.5–18.5%	1,6-Dimethyl-4-isopropyltetralin 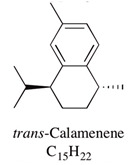	Major constituent of mānuka oil; contributes to insecticidal, antiseptic, bactericidal, analgesic and anti-inflammatory properties. Antibacterial effect against *S. aureus* and MRSA was shown.	[12,21]
δ-cadinene	0.9–6.9%	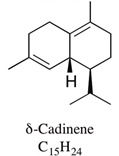	Pesticidal effects against mosquito have been shown for constituents isolated from *Kadsura heteroclita* leaf oil.	[22]
Cadina-1,4-diene	0.1–5.9%	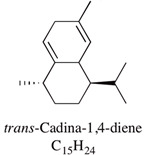	Not reported	
Cadina-3,5-diene	3.0–10.0%	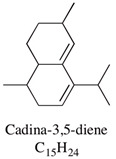	Not reported	
Flavesone	0.7–5.8%	2,2,4,4-Tetramethyl-6-(3-methylbutanoyl)cyclohexane-1,3,5-trione 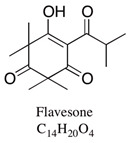	Antiviral properties.	[23]
α-copaene	4.3–6.5%	(1R,2S,6S,7S,8S)-8-isopropyl-1,3-dimethyltricyclo[4.4.0.02,7]dec-3-ene 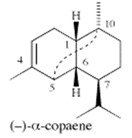 C_15_H_24_	Enhances mating in male Mediterranean fruit flies; Lures for trapping Redbay ambrosia beetle (*Xyleborus glabratus*).	[24,25]
α-selinene	1.3–5.0%	7-Epi-alpha-Selinene 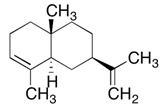 α-selinene C_15_H_24_	Insecticidal: retarding the growth of mosquito larvae.	[21]
α-terpineol	1–2%	2-(4-methylcyclohex-3-en-1-yl)propan-2-ol 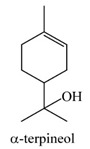 C_10_H_18_O	Antifungal effects; preservative for the postharvest storage of grapes and other fruits; has been shown to suppress the production of inflammatory mediators when sourced from Tea tree oil.	[26,27]
terpinene-4-ol	0.8–1.4%	4-methyl-1-propan-2-ylcyclohex-3-en-1-ol 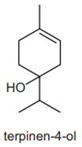 C_10_H_18_O	Spasmolytic activity; anti-inflammatory properties have been characterized in constituent isolated from *Melaleuca alternifolia*.	[28]

* IUPAC names were obtained from https://pubchem.ncbi.nlm.nih.gov/compound/; Chemical structures were obtained from https://pubchem.ncbi.nlm.nih.gov/.

**Table 2:** Antibacterial activity of Mānuka oil.

Organism	Method of Analysis	%(vol/vol) (µg/mL)		Relevance	Ref.
		^#^ MIC	* MBC		
***Gram positive bacteria***					
*Atopobium vaginae*	Broth microdilution	0.001	0.001	Vaginal infections, pre-term birth and neonatal infections	[53]
*Bacillus subtilis*	Broth microdilution	0.03	0.50	Intestinal bacteria	[35]
*Bacteroides vulgatus*	Broth microdilution	0.001	0.001	Vaginal infections, pre-term birth and neonatal infections	[53]
*Lactobacillus plantarum*	Broth microdilution	12.5	Not reported	Oral probiotic	[52]
H_2_O_2_-producing *lactobacilli and* non H_2_O_2_-producing *lactobacilli*	Broth microdilution	0.075	0.075	Vaginal bacteria	[53]
*Listeria monocytogenes*	Two-fold serial dilution	0.414	Not reported	Foodborne pathogen	[49]
*Gardnerella vaginalis*	Broth microdilution	0.001	0.001	Vaginal infections, pre-term birth and neonatal infections	[53]
*Propionibacterium acnes* ATCC 11827	Broth microdilution	0.055	Not reported	Acne	[41]
*Propionibacterium acnes*	Broth microdilution	0.211	0.25	Acne	[43]
*Staphylococcus aureus*	Two‘-fold serial dilution	0.535	Not reported	Foodborne pathogen	[49]
*S. aureus* strains	Two-fold serial dilution	0.513	Not reported	Multiple clinical manifestations in humans	[36]
Methicillin-resistant *S. aureus*	Broth microdilution	0.03	1.0	Skin infections, pneumonia, sepsis, surgical site infections	[35]
*Streptococcus agalactiae*	Broth microdilution	0.001	0.001	Meningitis; sepsis	[53]
*Staphylococcus epidermidis* ATCC 2223	Broth microdilution	1.40	Not reported	Acne	[41]
*S. intermedius*	Broth microdilution	0.0581	Not reported	Foodborne pathogen	[49]
*S. sorbinus*	Broth microdilution	0.048	Not reported	Oral pathogen	[36]
*S. sorbinus* 6715	96-well liquid culture microdilution	0.13	0.25	Oral pathogen	[38]
*S. sorbinus* B13	96-well liquid culture microdilution	0.25	0.25	Oral pathogen	
*S. mutans* JC2	96-well liquid culture microdilution	0.25	0.25	Oral pathogen	
*S. mutans* ATCC 25175	Two-fold microdilution	6.2	Not reported	Oral pathogen; dental caries	[52]
Vancomycin-resistant *Enterococcus faecalis* (VRE)	Broth microdilution	0.0064	Not reported	Sepsis; infection of open wounds	[45]
***Gram negative bacteria***					
*Actinobacillus actinomycetemcomitans (now known as Aggregatibacter actinomycetemcomitans) strains Y4, ATCC 29523, 29524, 33384*	96-well liquid culture microdilution	0.03	0.13	Oral pathogen	[38]
*Escherichia coli*	Broth microdilution	>4	>4	Intestinal bacteria; opportunistic pathogen	[35]
*E. coli* antibiotic and multidrug resistant strains	Two-fold microdilution	1–4	2–4	Hospital-based infections	[50]
*Fusobacterium nucleatum* ATCC 25586 *strains*	Broth microdilution	0.03	0.03	Periodontal disease; dental caries	[50]
*H. pylori*	Broth microdilution	Not reported	0.4	Gastritis, gastric ulcers and gastric cancer	[46]
*Klebsiella pneumoniae spp.* antibiotics and multidrug resistant isolates	Microdilution	2–4	2–8	Hospital-based infections; opportunistic pathogen	[50]
*Porphyromonas gingivalis* ATCC 33277, W50 and Su63	96-well liquid culture microdilution	0.03	0.06	Oral pathogen	[38]
*P. gingivalis* ATCC 53977	96-well liquid culture microdilution	0.03	0.03	Oral pathogen	[38]
*Pseudomonas aeruginosa* antibiotic and multidrug resistant isolates	Two-fold microdilution	≥8	≥8	Burn wound infections, sepsis	[50]
*Proteus mirabilis*	Two-fold microdilution	1–4	2–8	Hospital based infections	
*Salmonella typhimurium*	Two-fold serial dilution	0.00236	Not reported	Foodborne bacteria	[49]
*S. flexneri*	Two-fold serial dilution	0.00653		Foodborne bacteria	[49]
*S. sonnei*	Two-fold serial dilution	0.00697		Foodborne bacteria	[49]
*Serratia marcescens*	Broth microdilution	≥4	≥4	Opportunistic pathogen	[35]

* MIC: Minimum inhibitory concentration: minimum dose required to inhibit growth of bacteria; ^#^ MBC: Minimum bactericidal concentration: minimum dose required to kill bacteria.

**Table 3:** Antifungal activity of Mānuka oil.

Organism	* MIC (% *v*/*v*)	^#^ MFC (% *v*/*v*)	Relevance	Ref.
*Malassezia furfur*	1.56	Not reported	Pityriasis versicolor and Pityrosporum folliculitis	[37]
*Trichosporon mucoides*	1.56	Not reported	Opportunistic pathogen	
*Candida albicans*	3.13	Not reported	Opportunistic pathogen	
*Candida tropicalis*	3.13	Not reported	Opportunistic pathogen	
*Candida albicans*	0.015	0.015	Candida vulvovaginitis infections	[53]
*Candida glabrata*	0.010	0.010	Vaginal candidiasis	

* MIC: Minimum inhibitory concentration; ^#^ MFC: Minimum Fungicidal concentration. Both values were determined using microdilution assays.

**Table 4:** Antiparasitic or insecticidal effect of Mānuka oil.

Organism	Method	Lethal Effect	Clinical Significance	Ref.
**Acaricidal activity**				
*Dermanyssus gallinae*	Contact assay	LC_50_: 0.02 to 0.03, LC_90_:0.05 to 0.07 LD_99_: 0.10 mg/cm^2^	Poultry red mite	[58,59]
*D. farinae*	Fabricated disc method	LD_50_: 0.54 μg/cm^2^	House dust mite	[20]
*D. pteronyssinus*		LD_50_: 0.67 μg/cm^2^	House dust mite	
*Tyrophagus putrescentiae*		LD_50_: 1.21 μg/cm^2^	Stored product mite	
*Sarcoptes scabei*	Contact assay	LT_50_: 60 min for 5% solution LT_50_: 30 min for 10% solution	Human scabies mites	[14]
*Drosophila suzukii*	Contact assay	0.60 μg/mL for males and 1.10 for females	Fruit fly pest	[60]
*Aedes aegypti* (Linnaeus) larvae	Larvicidal bioassay	LC_90_: 66.62	Malaria	[21,61]
**Repellent effects**				
*Dermanyssus gallinae (De Geer)*	Fumigant assay	80–84%	Poultry red mite	[62]
*Dermanyssus gallinae*	Fumigant assay	80%	Poultry red mite	[8,59]
*Sarcoptes scabei*	Fumigant assay	80%	Human scabies mites	[14]

**Table 5:** Cytotoxicity of Mānuka oil in vitro.

Cell Line	Assay	Test	* TC_50_	Control	Ref.
RC-37 cells (African green monkey kidney cells)	Neutral red assay after treatment for 72 h	β-pinene	0.006%	1% ethanol	[68]
RC-37 cells (African green monkey kidney cells)	Neutral red assay after treatment for 96 h	Mānuka oil Leptospermone	0.0042% 0.04%	2.6% ethanol	[68]
THP-1 (monocyte/macrophage cell line)	XTT cell viability assay after 48 h of treatment	0.1–10% dissolved in DMSO	No toxicity	DMSO	[37]
Vero (African green monkey kidney cells)	Neutral red assay 96 h after treatment	Mānuka oil (0.001% to 1%) Leptospermone	0.0042% 0.04%	1% ethanol	[69]
HUVEC (Human umbilical vein endothelial cells)	Cell Titre Assay	Mānuka oil	~0.4%	No treatment	[38]

* TC_50_; the concentration of drug that reduces the number of viable cells by 50%.

## Abbreviations

ECHA	European Chemicals Agency
MIC	Minimum inhibitory concentrations
MBC	Minimum Bactericidal Concentration
EINECS	European Inventory of Existing Commercial chemical Substances
% *v/v*	volume per volume
VRE	vancomycin-resistant *Enterococcus faecalis*
MRSA	methicillin-resistant *S. aureus*
ATCC	American Type Culture Collection
EDTA	Ethylenediaminetetraacetic acid
LC_50_	lethal concentration required to kill 50% of the parasites
LD_50_	dose required to kill half the members of a tested population after a specified test duration
LPS	lipopolysaccharide
TNF-α	Tumor necrosis factor alpha
IL	Interleukin
UV	ultraviolet
HSV	Herpes simplex virus
IC_50_	half maximal inhibitory concentration
US	United States of America
EU	European Union
CTFA	Cosmetic, Toiletry and Fragrance Association
HUVEC	human umbilical vein endothelial cells
LDM	Living Dermal Matrix
3D	three-dimensional
ARTG	Australian Register of Therapeutic Goods.

## Appendix A

**Table A1:** Search Strategy for PubMed and Embase (Via Scopus).

PubMed	Search Strategies
#1	(“leptospermum”[MeSH Terms] OR “leptospermum”[All Fields]) OR “leptospermums scoparium”[All Fields]) OR (“manuka”[All Fields] AND “oil”[All Fields])) OR ((“tea tree oil”[MeSH Terms] OR “tea tree oil”[All Fields] NOT (honey) [All Fields])
#2	((“antifungal”[All Fields]) OR ((((((“anti-infective agents” OR “anti-infective agents”[MeSH Terms]) OR “anti-infective agents”[All Fields]) OR “antimicrobial”[All Fields]) OR “anti-microbial”[All Fields]) OR “antimicrobials”[All Fields] OR anti-inflammatory OR anti-inflammatory OR antiviral OR anti-viral OR “anti-viral agents”))
#3	#1 AND #2
**Embase (via Scopus)**	
#1	leptospermum OR “leptospermums scoparium” OR “manuka oil” OR “tea tree oil” OR “tea tree” NOT “honey”
#2	“anti-infective agents” OR antibacterial OR “antibacterial agent” OR “anti-infective agents” OR “anti-infective agents” OR “antimicrobial” OR “anti-microbial” OR “antimicrobials” OR anti-inflammatory OR anti-inflammatory OR antiviral OR anti-viral OR “anti-viral agents”
#3	#1 AND #2
